# Disseminated Tuberculosis Involving Lung, Peritoneum, and Endometrium in an Immunocompetent 17-Year-Old Patient

**DOI:** 10.7759/cureus.9081

**Published:** 2020-07-09

**Authors:** Alex Chua, Justin Nichols, Jonathan C Li, Cynthia E Flynn, Kristen Facciolo

**Affiliations:** 1 Internal Medicine-Pediatrics, ChristianaCare, Newark, USA; 2 Internal Medicine-Pediatrics Residency Program, ChristianaCare, Newark, USA; 3 Internal Medicine-Pediatrics Residency Program, University of Pittsburgh Medical Center, Pittsburgh, USA; 4 Sidney Kimmel Medical College, Thomas Jefferson University, Philadelphia, USA; 5 Pathology, ChristianaCare, Newark, USA; 6 Internal Medicine: Infectious Disease, ChristianaCare, Newark, USA

**Keywords:** disseminated tuberculosis, genital tuberculosis, pregnancy, internal medicine-pediatrics, pediatrics, internal medicine, infectious disease, global health, spontaneous abortion, obstetrics and gynecology

## Abstract

A 17-year-old Guatemalan female with a recent history of spontaneous abortion requiring dilation and curettage at 16 weeks' gestation presented two weeks post-procedure to a pediatric hospital for three days of worsening generalized abdominal pain, diarrhea, fevers, and cough. The patient's vital signs showed hypoxia, tachypnea, tachycardia, and hypotension; she was alert and oriented with a thin body habitus and suprapubic abdominal tenderness without rebound, guarding, or hepatosplenomegaly. She had no crackles, rales, or wheezing on lung examination. Labs revealed neutrophilic leukocytosis, acute kidney injury, transaminitis, and coagulopathy. Pelvic ultrasound demonstrated a septated pelvic fluid collection with an endometrial thickening. CT abdomen and pelvis showed significant nodular omental thickening and ascites. CT angiogram of the chest demonstrated an apical lung cavity and bilateral micro-nodularity without lymphadenopathy. Due to concern for septic shock secondary to endometritis, the patient was started on broad-spectrum antibiotics and intubated for acute hypoxic respiratory failure.

Repeat dilation and evacuation revealed degenerative first trimester products of conception and necrotizing granulomatous endometritis with *Mycobacterium tuberculosis* (*M. tuberculosis*) bacteria. Paracentesis indicated tuberculosis (TB) in ascites fluid, and bronchoalveolar lavage (BAL) showed pulmonary TB. Human immunodeficiency virus (HIV) screen and serum QuantiFERON®-TB Gold testing were negative. Rifampin, isoniazid, pyrazinamide, and ethambutol (RIPE) therapy was initiated alongside piperacillin-tazobactam for the treatment of both disseminated TB and septic abortion. She was extubated with hemodynamic stability, but fevers persisted. Repeat fallopian tube fluid sampling after five weeks of RIPE indicated numerous acid-fast bacilli. The patient’s septic clinical picture clouded her TB diagnosis as it appeared unusual that a healthy 17-year-old would concurrently have a septic abortion and disseminated TB; the lack of lymphadenopathy on CT scan also contributed to diagnostic uncertainty. Among patients from endemic regions, TB is a cause of spontaneous abortion. Conversely, during pregnancy, progesterone suppresses the T-helper 1 (Th1) proinflammatory response and increases susceptibility to TB. Peripartum women are at higher risk for disseminated TB, and postpartum women are twice as likely to experience reactivation of latent TB than nonpregnant women. Disseminated TB must be considered in pregnant adolescents presenting with appropriate clinical characteristics and imaging findings.

## Introduction

Disseminated tuberculosis (TB) involving multiple organs is rare among patients in developed countries, particularly immunocompetent patients [1,2]. However, pregnancy is a risk factor for reactivation and/or dissemination of TB [3,4]. In developing countries, TB often causes pregnancy loss, and disseminated disease should be considered as an underlying etiology in systemically ill immigrant females after a spontaneous abortion [5,6].

## Case presentation

A 17-year-old pregnant female, a recent Guatemalan immigrant with no known past medical history, presented to an outside adult hospital with abdominal pain at 16 weeks' gestation. She was found to have had a spontaneous abortion and underwent a dilation and curettage. Histology of amniotic sac contents showed a phenotypically male fetus with no identifiable gross abnormalities, immature internal organs with mild autolytic changes, acute chorioamnionitis, and a trivascular umbilical cord. Placental histology showed fragments of immature placental tissue and necrotic decidua.

**Figure 1:**
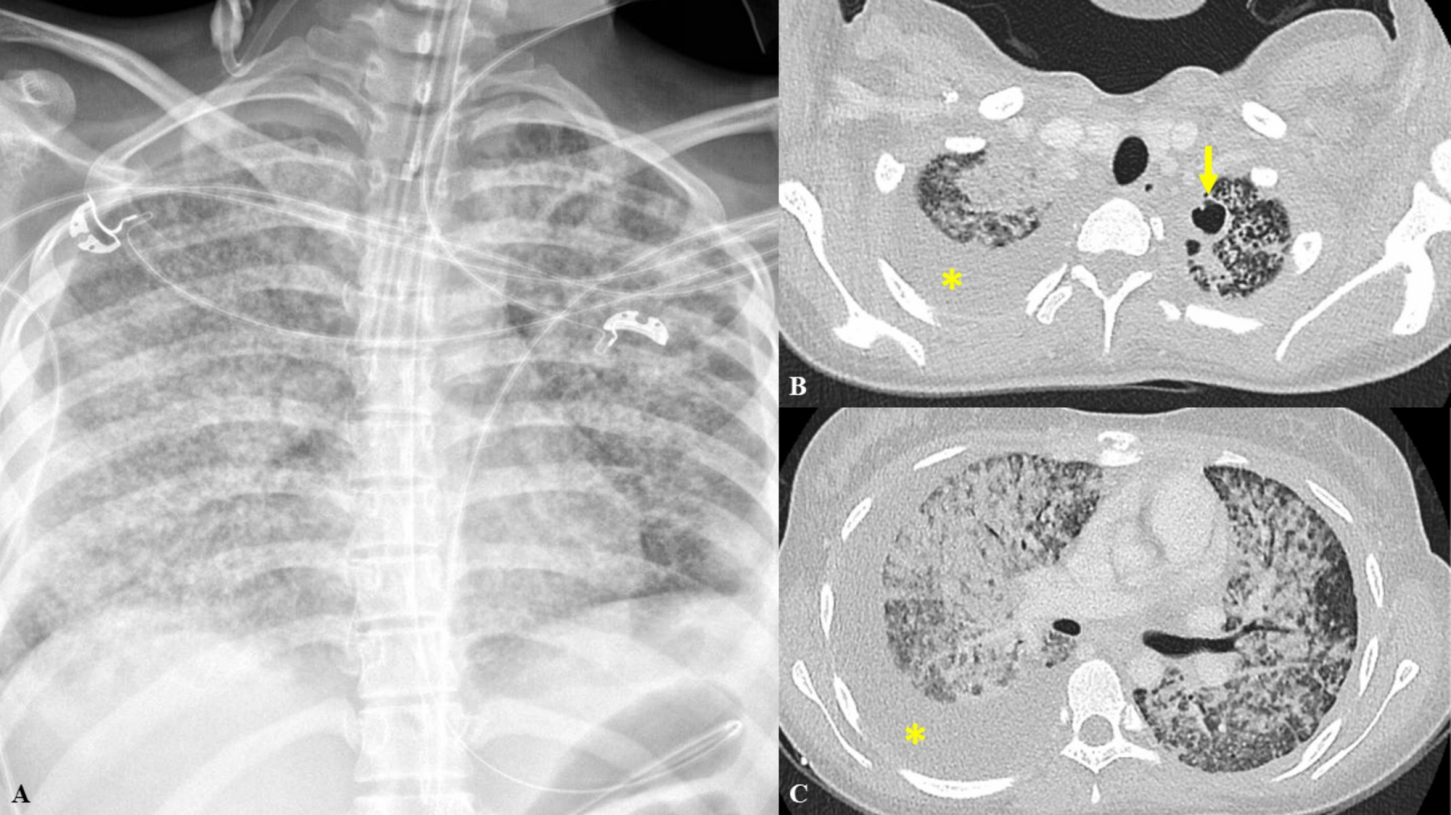
Chest imaging A) Post-intubation chest X-ray showing “severe bilateral lung opacities in a coalescing miliary type appearance. Small bilateral effusions.” B) Select CT Chest image showing “...a thick-walled pleural-based cavitary lesion medial left lung apex measuring 17 x 14 mm…”. Cavitary lesion identified by the arrow. C) Select CT chest image showing “...coalescence of innumerable parenchymal micronodules… Focal pleural-based mass-like opacity lateral left lower lobe measuring 26 x 14 mm…”. Pleural opacity identified by the asterisk CT: computed tomography

**Figure 2:**
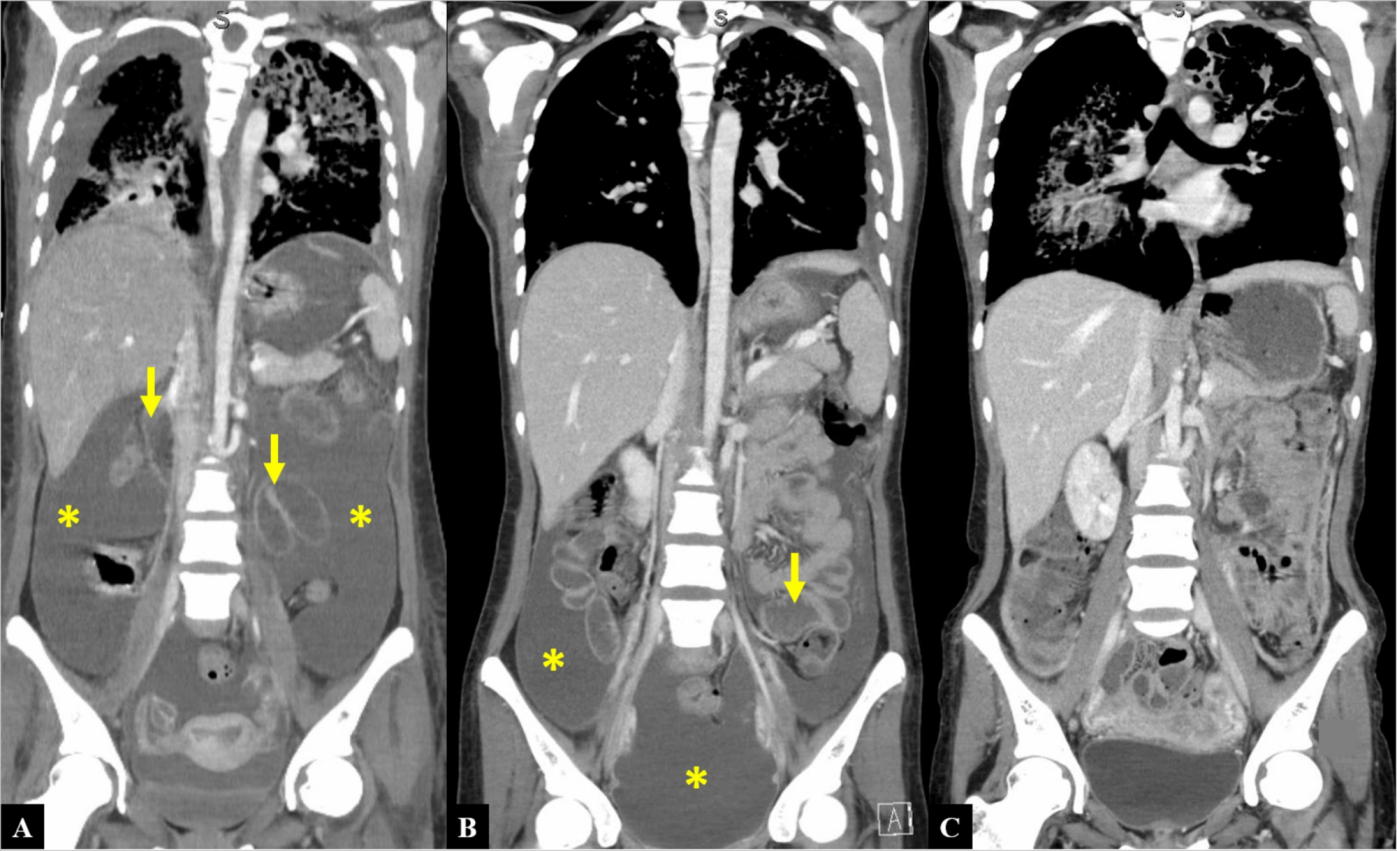
CT of the chest, abdomen, and pelvis at different clinical time points A) Depicts “...large amount of abdominal ascites present with areas of omental thickening... Mild dilatation of small bowel loops with fluid and gas…”. Ascites identified by asterisks. Bowel look dilations identified by arrows. B) Two weeks from initial CT. C) Eight weeks from initial CT CT: computed tomography

**Figure 3:**
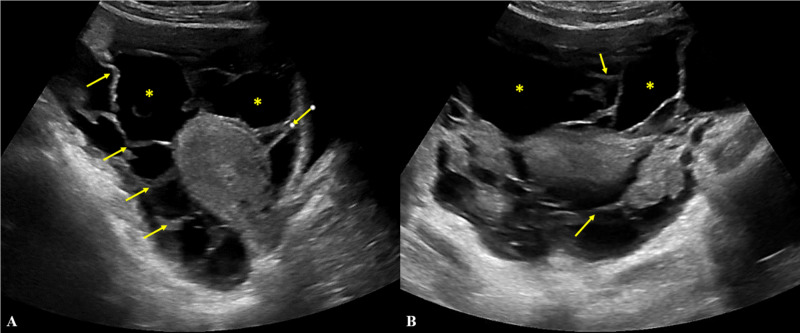
Transabdominal pelvic ultrasound with selected sagittal (A) and axial (B) views of the uterus This study was significant for “complex septated fluid throughout the pelvis”. Free fluid is identified by asterisks. Septations identified by arrows

Two weeks later, she presented to a pediatric hospital for three days of worsening abdominal pain, diarrhea, nausea, fever, and cough. Physical exam was remarkable for thin appearance, tachypnea, and suprapubic abdominal tenderness without rebound or guarding. Initial labs revealed leukocytosis with neutrophil predominance, acute kidney injury, and synthetic liver dysfunction. CT angiogram of the chest obtained for hypoxemia demonstrated an apical lung cavity with extensive interstitial thickening and micro-nodularity throughout both lungs (Figure 1), while CT abdomen and pelvis showed small bowel thickening, significant nodular omental thickening, and ascites (Figure 2). A pelvic ultrasound revealed a complex, septated fluid collection in the pelvis, endometrial thickening, and right upper quadrant ascites (Figure 3). Notably, no lymphadenopathy was seen on CT scan. Due to concern for septic shock secondary to endometritis, the patient was started on broad-spectrum antibiotics and subsequently transferred to an adult tertiary center where she required intubation for acute hypoxic respiratory failure.

**Figure 4:**
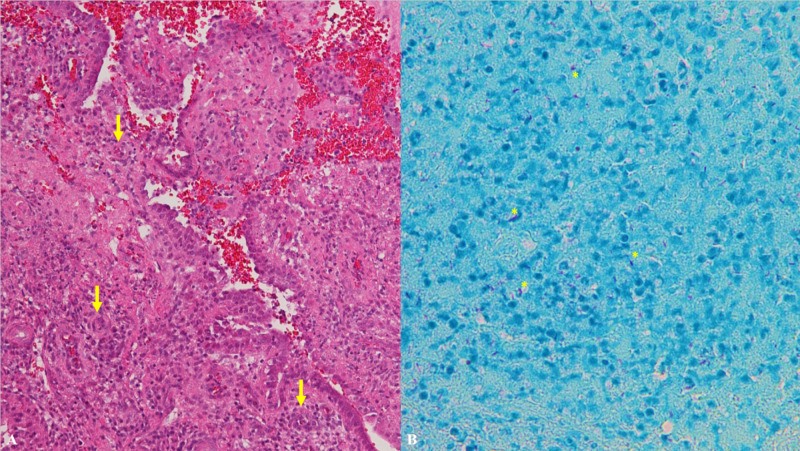
Histologic analyses of tissue removed from repeat dilation and evacuation A) Histology of endometrial curettage depicting necrotizing granulomatous endometritis. Select granulomas identified by arrows. B) Endometrial curettage sample with positive acid-fast staining organisms (pink); stained with Kinyoun acid-fast stain. Select acid-fast staining organisms identified by asterisks

**Figure 5:**
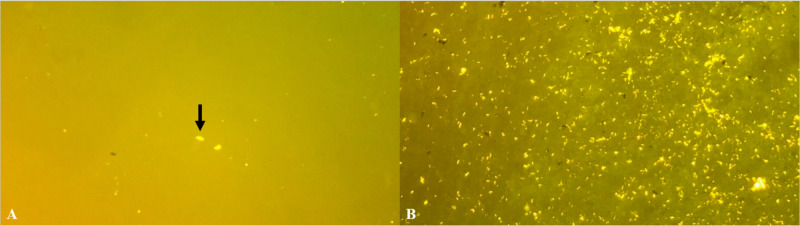
A) Peritoneal fluid with positive acid-fast staining organisms. B) Endometrial swab sample with numerous positive acid-fast staining organisms; stained with auramine-rhodamine stain

Although TB was on the differential at transfer, lack of lymphadenopathy on imaging was considered unusual for this presentation; however, a repeat dilation and evacuation revealed necrotizing granulomatous endometritis with moderate numbers of *Mycobacterium tuberculosis *(M. *tuberculosis*) bacteria, and degenerative first trimester products of conception (Figure 4). Bedside paracentesis also demonstrated *M. tuberculosis* in ascites fluid (Figure 5A). Endometrial swab performed days following dilation and evacuation revealed numerous *M. tuberculosis *organisms(Figure 5B). Bronchoscopy/bronchoalveolar lavage (BAL) was performed, which revealed pulmonary *M. tuberculosis*. Human immunodeficiency virus (HIV) screen and a serum QuantiFERON®-TB Gold testing were both negative. Rifampin, isoniazid, pyrazinamide, and ethambutol (RIPE) therapy was initiated, and the patient completed a three-week course of piperacillin-tazobactam given the initial septic presentation with retained products of conception.

**Figure 6:**
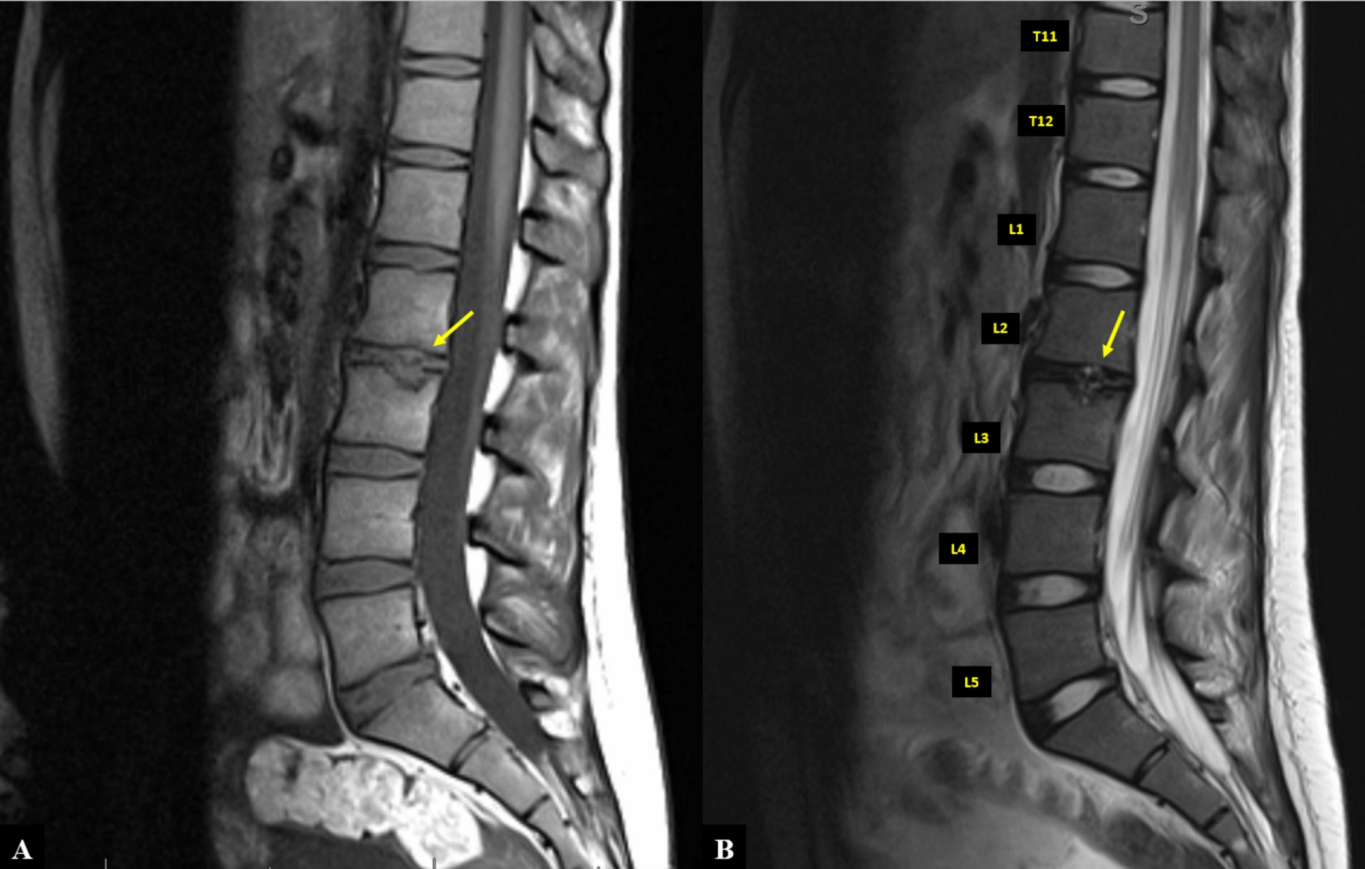
MRI of the lumbar spine A) T1-weighted image. B) T2-weighted image. Arrows indicate the location of the Schmorl's nodule MRI: magnetic resonance imaging

**Figure 7:**
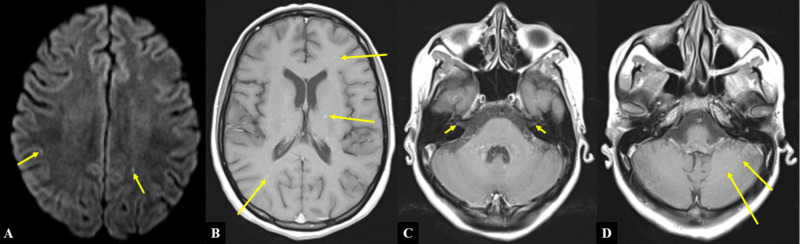
MRI of the brain A) Diffusion-weighted image of bilateral cerebral hemispheres. B-D) T1-weighted images of selected coronal sections in superior-to-inferior order. Arrows indicate locations of select hyperintense lesions. Note that not all lesions are identified by the arrows MRI: magnetic resonance imaging

Although the patient hemodynamically stabilized and was successfully extubated, despite two weeks of RIPE, she continued to spike fevers and experience night sweats. A complete MRI spine was performed to screen for spinal involvement and showed L2-L3 disc desiccation and a Schmorl’s nodule, but no active infection (Figure 6). An MRI brain revealed two restricted diffusion lesions concerning for septic emboli, and subtle linear enhancement along the 7th, 8th, and cisternal trigeminal nerves, suspicious for early basilar meningitis (Figure 7). A lumbar puncture was performed with cerebrospinal fluid studies, which came back negative for active TB. Repeat fallopian tube fluid sampling after nearly five weeks of RIPE therapy continued to demonstrate numerous acid-fast bacilli.

## Discussion

In 2018, an estimated 10 million people contracted TB worldwide, with 15% of cases demonstrating extrapulmonary TB [1]. Concurrently, in the US, the Centers for Disease Control and Prevention (CDC) reported approximately 9,000 cases of TB, with 20% having extrapulmonary manifestations. The most common extrapulmonary sites were lymphatic (36.9%), pleural (16.9%), bone or joint (9.6%), peritoneal (5.8%), genitourinary (4.8%), meningeal (3.8%), and laryngeal (0.2%) [2]. Once TB has infected a primary site, typically the lungs, it can spread in a lympho-hematogenous manner and has the potential to seed any vascular bed [7]. The greatest risk factor for developing disseminated TB is an immunocompromised/suppressed state [7,8].

Pregnancy, from a simplified perspective, represents an immunosuppressed state that allows for embryologic implantation and fetal development without maternal (host) rejection [9]. Progesterone in pregnancy suppresses the T-helper 1 (Th1) proinflammatory response, which increases susceptibility to infectious diseases such as influenza, varicella, and TB [3,4,10]. This risk is highest during the third trimester [11]. As a result, pregnancy can mask symptoms of TB [5,12]. Th1-suppression remits during the postpartum period resulting in the manifestation and/or exacerbation of clinical disease [3,4,12]. Therefore, peripartum women are at high risk for asymptomatic, disseminated TB, and postpartum women are twice as likely to experience reactivation of latent TB as nonpregnant women [4,12]. Both interferon-gamma release assays (IGRA) and tuberculin skin tests are dependent on an intact Th1 immune response. False-negative results increase during pregnancy due to suppressed Th1 response [13].

Globally, as reviewed by Ghosh et al., TB is responsible for infertility in 1-18% of cases with higher rates in developing countries [6]. Among patients with reproductive tract TB, the incidence of infertility ranges from 10-85% [6]. The most commonly affected organs are the fallopian tubes (92-100%), the ovaries (10-30%), the endometrium (50%), and less commonly the cervix (5%) [6]. Early TB may be difficult to differentiate from pregnancy due to similar generalized symptoms including malaise and fatigue [6]. Specifically, reproductive tract TB is difficult to identify, as the most common clinical presentation is simply infertility [14].

We hypothesize that our patient had asymptomatic active TB during her pregnancy as she did not have any documented symptoms prior to presentation. Both the inflammatory milieu of TB and direct infection of the female genital tract leads to a higher rate of spontaneous abortion [3-6]. Dissemination or activation likely occurred due to the immunologic changes she experienced during pregnancy, and progressively became more symptomatic following spontaneous abortion as progesterone levels normalized. Cheng et al. have described a similar cohort of patients who developed peripartum TB in an immunorestitution disease-like manner [12]. Despite a negative IGRA, it is important to acknowledge that these tests can be falsely negative 8-16% of the time in active TB, and also in the setting of pregnancy [13,15]. The patient’s septic clinical picture with retained products of conception clouded her TB diagnosis over the first two days of admission as it appeared unusual that a previously healthy 17-year-old would concurrently have sepsis from retained products of conception and disseminated TB.

## Conclusions

TB is a common cause of spontaneous abortion among fertile females in developing countries. Immunologic changes during pregnancy can affect testing and spur asymptomatic disease. Clinical suspicion for TB should remain high among patients from endemic regions, even in patients presenting with a seemingly unrelated gynecologic problem, such as spontaneous abortion. Pregnant patients from endemic regions should be screened for asymptomatic disease with IGRA. However, a negative result should be interpreted cautiously with respect to clinical history, signs, and symptoms.
